# Tension-free vaginal tape versus lata fascia sling: The importance of transvulvar ultrasound in the assessment of relevant anatomical parameters in treatment of women with stress urinary incontinence

**DOI:** 10.4103/0970-1591.45539

**Published:** 2009

**Authors:** Frederico Teixeira Brandt, Felipe Lorenzato, Carla Daisy Costa Albuquerque, Agostinho de Sousa Machado Junior, Amanda de Carvalho Poça, Raíssa Almeida Viana

**Affiliations:** Urinary Incontinence Research Unit (URIU) at the Universidade Federal de Pernambuco (UFPE), Avenida Dezessete de Agosto, 2475 - Apartamento 2801, Recife- Pernambuco, 52061-540 - Brazil

**Keywords:** Fascia Lata Sling Procedure, stress urinary incontinence, tension-free vaginal tape, transvulvar or perineal ultrasound, urethrovesical junction mobility and proximal urethra length

## Abstract

**Objective:**

To describe the relevance of transvulvar ultrasound in the assessment of anatomical differences induced by the lata fascia sling (LFS) and tension-free vaginal tape (TVT) procedures.

**Materials and Methods:**

Forty women with stress urinary incontinence (SUI), aged 30 to 60 years, have been treated with either LFS (20 patients) or TVT (20 patients). The transvulvar ultrasound of the urethrovesical junction (UVJ) and proximal urethra (PU) has been used as the main investigational tool both pre- and post-operatively. The studied parameters were the vertical (VUVJD) and horizontal (HUVJD) UVJ distances, the pubourethral distance (PUD) and the PU length.

**Results:**

The VUVJD did not vary significantly after the LFS surgery (*P*=0.10). The PUD became shorter (*P*=0.001) and the HUVJD became shorter only at rest (*P*=0.03) after the correction by LFS. The TVT procedure has led to shortening of the VUVJ displacement (*P*=0.0005) and of the PU length (*P*=0.02).

**Conclusions:**

The transvulvar ultrasound was of utmost importance in the demonstration that both the LFS and TVT surgical procedures elongate the PU, even though the LFS technique does it more efficiently. The LFS technique focus more on shortening the PUD and the TVT procedure focus more on the correction of the vertical UVJ displacement.

## INTRODUCTION

Female urinary incontinence affects millions of women. Its prevalence rates are considerably high worldwide, reaching 28% in some European countries and 37% among adult North American women, according to a recent national research undertaken in USA.[1] The relevance of this problem is reflected in an anual expenditure of about 15 billion dollars in treatment costs and also in the great impact on patients’ quality of life, causing low self-esteem, embarrassing situations and social isolation. Only 25% of the women who suffer from some degree of urinary incontinence and about 50% of patients with significant incontinence seek a doctor's advise to solve their problem.[1–6]

Different surgical procedures using the sling technique have been developed with the aim of restoring basic urinary continence principles, yet always attempting the minimization of complications. These principles are: avoidance of sphincteric insufficiency or obstruction, proper localization and provision of support to the urethrovesical junction (UVJ) and proximal urthra (PU) so that they can effectively respond to increases in intra-abdominal pressure. The main basic concept is to make sure the physiologic mechanisms of micturition will not be disturbed by either over or under suspension of the bladder neck.[7–10]

Although developed in 1907, the sub-urethral sling technique has only started to be more frequently used for treatment of stress urinary incontinence (SUI) in the last decades. Historically the sub-urethral sling technique has used either autologous, heterologous or synthetic materials as a sling passing behind the pubis, across the vagina, and underneath the PU, in such a way to offer adequate support to the posterior urethral segment. This procedure has become very well accepted in recent years and its success rates vary from 70 to 100%. The sub-urethral sling is being considered as a first line of treatment for types I and II SUI, where the UVJ descends beyond pubic symphysis during stress.[7–10]

Initially, not much importance has been given to an objective assessment of UVJ and PU before and after surgical treatment of female SUI. The focus has ever been short and long term clinical efficacy. However, in recent years more and more attention has been driven to such objective measurements and to a post-operative quality control assessment of anatomical parameters.[8,10]

Since the last decade researchers have been showing progressive interest on UVJ and PU anatomical parameters, especially on the vertical UVJ mobility.[11–17] With this new concept, the assessment of the UVJ mobility in the vertical axis using the transvulvar or perineal ultrasound as a primary tool for measurement of key parameters has been gaining a broader acceptance.

Although the sling surgical technique for correction of SUI has been widely used internationally with good results, it is considered expensive for developing countries to implement it on a large scale. The main issue here is the need of heterologous materials either synthetic or not. The fact of the matter is that costs can turn its application too difficult or even impossible for lage scale use in developing countries.[18,19]

The utilization of homologous Lata Fascia Sling (LFS) has been reducing substantially the costs related to this surgery, making it viable for many more patients, especially the ones from less privileged regions.[12]

The present study aims at describing the anatomical changes induced by either LFS or Tension-Free Vaginal Tape (TVT) in the treatment of women with SUI, assessed by transvulvar ultrasound with the bladder practically empty.

## MATERIALS AND METHODS

This is a prospective longitudinal study undertaken at the Urinary Incontinecy Research Unit (UIRU) from the University Hospital and Clinics at Universidade Federal de Pernambuco (HC-UFPE) between August 2004 and January 2006.

The research protocol was reviewed and approved by the local Ethics Committee before the first voluntary was included. After an explanation in lay terms of all study details to eligible women, those who accepted to participate were invited to sign in an informed consent form.

Forty women with surgical indication for treatment of SUI were randomly included in this trial, 20 of them were assigned to surgical treatment with the Sling technique using the Lata Fascia and the other 20 were assigned to treatment with the TVT procedure.

The inclusion criteria were: women with a clinical diagnosis of SUI requiring surgical treatment, age between thirty and sixty years, vertical UVJ descent over 9 mm as assessed by transvulvar ultrasound, absence of other diseases that could cause urinary incontinence, no previous surgery involving either the urinary bladder, urethra or vagina. The exclusion criteria were: development of an acute disease that could turn the surgical procedure unviable, pregnancy and patient's withdrawal of consent, which have just not occurred.

All patients answered to a standard general anamnesis and to a detailed urogynecological questionnaire. Then they were submitted to a thorough physical examination including a complete urogynecological evaluation. The ultrasound parameters were assessed before and 30 days after the surgery. All participants were examined by a single ultrasound expert in the transvulvar approach, who had more than 5 years experience in procedure, thus minimizing both inter- and intra-observer biases.

**Figure 1:**
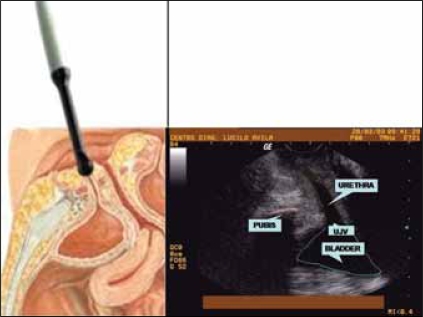
Illustration of a schematic diagram as compared to a transvulvar ultrasound view showing the main pelvic structures involved in female urinary continence

For the transvulvar ultrasound assessment of the UVJ, we used an ALOKA SSD 500 machine connected to a 7Mhz convex vaginal transducer. The measuments were taken from patients in the dorso lithotomy position with a barely empty bladder (having <50 ml of urine), as previously described.[14,17] To the tip of the ultrasound transducer ultrasound gel was added, which was covered by a glove and then more ultrasound gel was applied before probe was placed in contact with the perineum in a mid sagittal orientation. The transducer was positioned against the vulva right below the urethra, where the window view allows for a proper assessment of the urethra, bladder, UVJ and the pubis, as illustrated in Figure 1.

Measurements were taken both at rest and on strain (Valsalva's maneuver). The displacements were calculated by substraction of the value at rest from the one on strain. The zero level has been conventioned as the inferior pubic border. When the UVJ was positioned above the inferior pubic border both at rest and on strain, the measurement of its distance, either in the vertical or horizontal plane, was the distance in mm from the zero level to its present location and to it has been attributed a positive value (+). When the UVJ was located below the inferior pubic border both at rest and on strain, the measurement in mm of its distance, either in the vertical or horizontal plane, was calculated from the zero level to its present location and to it has been attributed a negative value (-). While measuring the UVJ displacement when the UVJ was initially above the inferior pubic border at the rest and went below it on strain, the final measurement was the sum of the positive value to the negative one disregarding the mathematical signaling standards.

**Figure 2:**
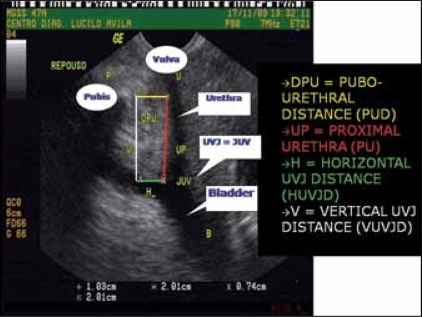
Transvulvar ultrasound image showing how the VUVJD, HUVJD, PUD and PU have been measured

The following parameters, as illustrated in Figure 2 and previously described,[14,17] have been measured:

1. Vertical UVJ Distance (VUVJD): the length in mm of a longitudinal straight line drawn from inferior pubic border to the point where it crosses a transversal line coming from the UVJ anterior margin. The VUVJD is described in Figure 2 in white.
2. Horizontal UVJ Distance (HUVJD): the length in mm of a transversal straight line drawn from the UVJ anterior margin to the point where it crosses a longitudinal line coming from the inferior pubic border. The HUVJD is described in Figure 2 in green.
3. Pubourethral Distance (PUD): the length in mm of a transversal straight line drawn from the urethra to the inferior pubic border. The PUD is described in Figure 2 in yellow.
4. Proximal Urethra (PU) length: the length in mm of a straight line drawn from UVJ anterior margin along the urehtra until the point where it is crossed by a transversal line coming from the inferior pubic border. The PU length is described in Figure 2 in red.

The criteria used to diagnose SUI with UVJ hipermobility have been suggested by Brandt *et al*. 2005. It is believed that the objective demonstration of vertical UVJ hypermobility (a VUVJ displacement over 9 mm) is a strong diagnostic indicator of SUI.[8,20]

The TVT surgical procedure was performed using a technique originally described by Ulmsten.[12] For the LFS it has been used an autologous piece of aponeurosis (measuring 2 × 6 cm) taken from the internal thigh side. After that, the sling was tailored to the specific needs and positioned to correct the SUI using a Pfannienstiel incision.

A month after the surgical procedure, a quality control ultrasound assessment of the anatomical parameters being studied was carried out in all enrollees.

The data were analyzed using the EPI-INFO 2002 version 1.0 statistical package. The following statistical tests have been used: the χ2 test was used for contingency tables, the 2-tailed Fisher' exact test was used for analysis of averages and variances when a cell on a 2X2 table scored less than 5. The level of statistical significance considered was a *P*-value < 0.05.

## RESULTS

A total of 40 women enrolled in the study. Their mean age was 50.3 ± 9.6 years, varying from 30 to 60 years.

The mean VUVJ ultrasound measurements both before and after the surgical procedures (either LFS or TVT) are described in Table 1.

**Table 1:** Mean measurements of the vertical urethrovesical junction distance (VUVJD) among patients with stress urinary incontinence before and a month after Lata Fascia Sling (LFS) or Tension-Free Vaginal Tape (TVT) procedure

VUVJD(mm)	At rest		On strain		Displacement	
	
	Before	After	Before	After	Before	After
LFS						
Mean	16.3	17.4	2.7	12.6	13.6	4.8
Standard deviation	5.7	4.8	11.5	5.7	11.4	5.8
Significance	ρ = 0.58		ρ = 0.10		ρ = 0.12	
TVT						
Mean	16.0	16.6	2.1	8.4	16.7	10.7
Standard deviation	4.6	3.2	15.3	9.6	7.7	7.3
Significance	ρ = 0.26		ρ = 0.28		ρ = 0.0005	

The mean HUVJ ultrasound measurements both before and after the surgical procedures (either LFS or TVT) are described in Table 2.

**Table 2:** Mean measurements of the horizontal urethrovesical junction distance (HUVJD) among patients with stress urinary incontinence before and a month after Lata Fascia Sling (LFS) or Tension-Free Vaginal Tape (TVT) procedure

HUVJD(mm)	At rest		On strain		Displacement	
			
	Before	After	Before	After	Before	After
LFS			
Mean	13.4	10.3	14.0	9.2	10.3	-1.1
Standard deviation	3.0	4.4	9.4	6.1	0.6	7.6
Significance	ρ = 0.03		ρ = 0.11		ρ = 0.60	
TVT						
Mean	11.4	11.0	16.4	15.4	7.2	5.6
Standard deviation	6.3	5.5	7.6	8.9	5.2	4.0
Significance	ρ = 0.33		ρ = 0.28		ρ = 0.18	

The mean PUD ultrasound measurements both before and after the surgical procedures (either LFS or TVT) are described in Table 3.

**Table 3:** Mean measurements of the pubourethral distance (PUD) among patients with stress urinary incontinence before and a month after Lata Fascia Sling (LFS) or Tension-Free Vaginal Tape (TVT) procedure[26]

PUD (mm)	At rest		On strain		Displacement	
			
	Before	After	Before	After	Before	After
LFS						
Mean	11.6	10.9	17.8	10.7	6.2	-0.2
Standard deviation	3.0	1.7	4.6	2.8	4.2	2.7
Significance	ρ = 0.38		ρ = 0.001		ρ = 0.001	
TVT						
Mean	13.4	10.9	18.3	14.4	5.7	4.8
Standard deviation	5.0	2.8	7.5	6.9	5.0	4.3
Significance	ρ = 0.24		ρ = 0.26		ρ = 0.43	

Amongst all findings, the most interesting one was the transvulvar ultrasound assessment of the PU length, which is described in Table 4.

**Table 4:** Mean measurements of the proximal urethra (PU) length among patients with stress urinary incontinence before and a month after Lata Fascia Sling (LFS) or Tension-Free Vaginal Tape (TVT) procedure

PU length (mm)	At rest		On strain		Displacement	
			
	Before	After	Before	After	Before	After
LFS						
Mean	16.8	18.5	1.1	10.7	−15.7	−7.9
Standard deviation	4.8	3.6	3.2	5.9	3.6	4.6
Significance	ρ = 0.28		ρ = 0.0 03		ρ = 0.001	
TVT						
Mean	15.3	17.3	3.7	8.4	13.1	9.2
Standard deviation	4.7	3.0	8.4	8.2	6.7	6.6
Significance	ρ = 0.30		ρ = 0.05		ρ = 0.02	

## DISCUSSION

The existence of more than one hundred types of surgical techniques developed to correct SUI has led us to think that the pathophysiology of this disease is not completely understood and, because of that, the surgical rationale is not totally known yet.[6,10,21]

Our findings suggest that some of the main anatomical parameters in the correction of SUI are the increase in length of the proximal urethra and the fixing of the UVJ hypermobility. These in turn should provide support to the proper maintenance of the proximal urethra pressure, thus restoring the adequate control of micturition.

There is a study line that defends urodynamic testing as the best complementary exam to diagnose SUI. On the other hand, transvulvar ultrasound for assessment of the UVJ and PU has been acquiring more and more acceptance in recent years. It is considered as an important investigational tool for objective documentation of anatomical parameters before and after surgery.[8,10,21]

Among the different types of surgical procedures to correct SUI, the old Sling technique has suffered changes based on new postulates of the TVT technique. The TVT results usually show advantages regarding the placement of the proximal urethra over other similar procedures. In spite of it, the majority of authors agree that urodynamic testing is the best to study patients with SUI, disregarding the relevance of important parameters that can be easily evaluated by transvulvar ultrasound.[8,10]

Although there is disagreement as to the ideal test to diagnose female SUI, there is reasonable agreement that the majority of these patients have changes to their anatomy, particularly to the UVJ and PU. Besides, approximately 95% of patients who complain of SUI have UVJ hypermobility and malfunctioning of the PU.[8,10–12,22]

In the last decade, there has been a clear progress in the body of evidence regarding factors that can influence the ultrasound examination of the UVJ and PU. As a matter of fact, bladder volume during examination as well as the intra-abdominal pressure have been shown to influence the UVJ mobility and the capacity of the PU to maintain urinary continence. Such findings are increasing doctors' interest and reliability in urogynecological ultrasound evaluations.[13,20]

Urodynamic testing evaluates only pre-operative parameters and tries to indirectly infer the concept of UVJ mobility. However, unlike the ultrasound, urodynamic testing cannot document objectively the anatomical position of the UVJ and the PU length, neither in a static or dynamic scenario.[8,10,12,14] These facts have led us to adopt the transvulvar ultrasound approach with a barely empty bladder as the investigational tool in the present research work.

The fascia of the recto-abdominalis muscle is frequently used but the superiority of the Lata fascia procedure is well recognized, particularly for less tissue injury, risks and complications, the possibility to obtain larger fascia sizes, shorter hospital stay. Therefore, for a more rapid return of patients to their usual activities.[19]

It was interesting to observe, through the eyes of transvulvar ultrasound, that the vertical UVJ displacement, considered one of the most important anatomic parameters in the maintenance of urinary continence, was only significantly modified after the TVT surgery [Table 1]. One has though to bear in mind to avoid overcorrection that may cause some degree of urinary obstruction.

Authors from different regions of the world, using a similar ultrasound approach (transvulvar or perineal), sometimes varying in bladder volume or patients position at examination, have described pre- and post-operative results regarding different surgical procedures aimed at correcting female SUI. Their findings regarding the vertical UVJ descent are in line with our observations.[4,8,10,15,23–25]

On the other hand, the LFS procedure has also corrected most patients’ SUI even though the elevation of the UVJ or decrease in the vertical UVJ distance and displacement were not significant [Table 1].

The horizontal UVJ measurements were not significantly affected by any of the surgical corrective techniques used [Table 2]. It is noteworthy that only the LFS procedure managed to bring the UVJ significantly closer to the pubis, although this shortening of the PUD has not been shown significant at rest [Table 3].

The transvulvar ultrasound evaluation has allowed us to observe that among our results, perhaps, the most important variable was the elongation of the proximal urethra both on strain and in relation to its displacement after the LFS procedure (more pronouncedly) as well as after the TVT correction of female SUI [Table 4]. Therefore, the physiological relevance of this surgically induced anatomical changes may be the restoration of the pressoric mechanism at the proximal urethra through an increase in its area due to its elongation, thus restoring urinary continence back again.

At post-operative day 30, we observed that both the surgical techniques (LFS and TVT) have led to the disappearance of the SUI symptoms in 85% of patients, which corroborates the data described in the literature.[9]

It is important to make it clear that short term cure rates are of limited valued and evaluation of long term prognosis has not been in the scope of the presente study. It is common sense that the assessment of cure rates for women with SUI should have a minimum 10 years follow-up.

Nevertheless, it is high time we started discussing more thoroughly the importance of the ultrasound examination in the post-operative period as a means of anatomical correction quality control. A cut-off point of 5 mm for the vertical UVJ displacement has been established as the average for young continent patients[14] and it also suggests an appropriate hallmark for satisfactory surgical correction of female SUI.[15] It is hypothesised that this treshold can also serve as a prognostic factor for recurrence of SUI, as postulated by other researchers.[16] As a matter of fact, this is one of our new study's goal in progress.

Our overall findings suggest that both the LFS and TVT procedures are equivalent regarding the augmentation of PU length and short term clinical results. One thing though, that deserves more studies with larger sample sizes, is the need for cost-effectiveness and cost-benefit analyses to compare both procedures and test the advantages of LFS regarding its low cost compared to TVT, which requires the use of synthetic materials. And finally, bear in mind that the LFS technique has been shown to offer a shorter surgical time and hospital stay.

## CONCLUSION

Transvulvar ultrasound assessment of the urethrovesical junction (UVJ) and proximal urethra is a very important tool for objective documentation of anatomical structures essentially involved in pathophysiologic mechanisms leading to female SUI. The characterization of vertical UVJ hypermobility and the length of the proximal urethra prior to surgical correction may be of use for post-operative quality control. In addition, the transvulvar ultrasound evualuation can be used to compar the impact different surgical approaches produce in the discussed parameters. For instance, in the present study we have observed that only the TVT procedure, compared to the LFS, managed to decrease the vertical UVJ displacement significantly. There were minor decreases in horizontal UVJ distances after both surgical procedures, although these were not significant. The LFS surgery managed to shorten the PUD significantly while the TVT procedure did not. As expected for the re-establisment of proper urinary continence, the proximal urethra was significantly elongated after both surgical procedures being compared.

In summary, the transvulvar ultrasound performed in women with a barely empty bladder has allowed for the observation that the TVT procedure has mainly elongated the proximal urethra and reduced the vertical UVJ displacement. On the other hand, the LFS has brought the UVJ closer to pubis and also elongated the proximal urethra but less pronouncedly than the TVT procedure. In other words, both TVT and LFS surgical procedures have allowed these key structures to be put back to where it used to be, thus restoring their proper functioning. The long term clinical implications of whether these post-operative anatomical differences would lead to distinct outcomes still remain to be shown.
